# Genomic integration and ligand-dependent activation of the human estrogen receptor α in the crustacean *Daphnia magna*

**DOI:** 10.1371/journal.pone.0198023

**Published:** 2018-06-08

**Authors:** Kerstin Törner, Tsuyoshi Nakanishi, Tomoaki Matsuura, Yasuhiko Kato, Hajime Watanabe

**Affiliations:** 1 Department of Biotechnology, Graduate School of Engineering, Osaka University, Yamadaoka, Suita, Osaka, Japan; 2 Laboratory of Hygienic Chemistry and Molecular Toxicology, Gifu Pharmaceutical University, Daigaku-nishi, Gifu, Gifu, Japan; 3 Frontier Research Base for Global Young Researchers, Graduate School of Engineering, Osaka University, Yamadaoka, Suita, Osaka, Japan; Massachusetts General Hospital, UNITED STATES

## Abstract

The freshwater crustacean *Daphnia* have a long history in water quality assessments and now lend themselves to detection of targeted chemicals using genetically encoded reporter gene due to recent progress in the development of genome editing tools. By introducing human genes into *Daphnia*, we may be able to detect chemicals that affect the human system, or even apply it to screening potentially useful chemicals. Here, we aimed to develop a transgenic line of *Daphnia magna* that contains the human estrogen receptor alpha (hERα) and shows a fluorescence response to exposure of estrogens. We designed plasmids to express hERα in *Daphnia* (EF1α1:*esr1*) and to report estrogenic activity via red fluorescence (ERE:*mcherry*) under the control of estrogen response element (ERE). After confirmation of functionality of the plasmids by microinjection into embryos, the two plasmids were joined, a TALE site was added and integrated into the *D*. *magna* genome using TALEN. When the resulting transgenic *Daphnia* named the ES line was exposed to Diethylstilbestrol (DES) or 17β-Estradiol (E2), the ES line could reliably expressed red fluorescence derived from mCherry in a ligand-dependent manner, indicating that an estrogen-responsive line of *D*. *magna* was established. This is the first time a human gene was expressed in *Daphnia*, showcasing potential for further research.

## Introduction

The crustacean *Daphnia*, as keystone species of freshwater ecosystems, have been used in water quality assessments for many decades [1,2]. High growth rate and high fecundity combined with cheap and easy rearing make them an excellent organism for screening. Recently, the sequencing of the *Daphnia pulex* and *Daphnia magna* genomes [3,4] has been completed and different methods for genomic integration of foreign genes like CRISPR/Cas [5] and TALEN-mediated knockin [6,7] have been successfully adapted in *D*. *magna*. In order to facilitate the expression of ectopic gene, an optimal mRNA structure in *D*. *magna* was also reported [8]. These progress of functional genomics allowed us for detecting one of arthropod hormones, ecdysteroid using a GFP reporter gene under control of ecdysteroid responsive elements in this species [9]. In addition, a candidate of an element responsive to another arthropod hormone, juvenile hormone, was integrated together with its reporter GFP gene into the genome [10]. These reporter constructs and transgenic lines have potential as tools to detect agonists of ecdysteroids and juvenile hormones that are widely used as pesticides, which can be used as a chemical sensor *Daphnia*.

Based on the progress of the development of chemical sensor *Daphnia*, we aimed to detect chemicals that affect the human system [11–12] by making sensor Daphnia. In this study, we aimed to develop estrogen sensor Daphnia by introducing human genes, as *Daphnia* hormonal system is different from human and estrogen system does not exist in Daphnia. Estrogens and estrogen like chemicals bind to estrogen receptors (ER), the resulting complex interacts with Estrogen Response Elements (ERE) on the target gene of genome and activate transcription of downstream genes [13–15]. In this study, we designed and integrated a plasmid to express the human estrogen receptor alpha (hERα) (EF1α1:*esr1*) and to report estrogenic activity via red fluorescence (ERE:*mcherry*) into the genome of *D*. *magna* using a set of previously established TALENs [6]. The resulting estrogen sensor line (ES line) reliably indicates presence of Diethylstilbestrol (DES), a synthetic estrogen, as well as that of 17β-Estradiol (E2), a natural estrogen. To our knowledge this is the first time a human gene has been successfully expressed in *Daphnia*, showcasing the potential to test the response of different human genes to environmental stimuli relatively directly, and therefore determine potential health impacts in both animals and humans.

## Results

**Fig 1:**
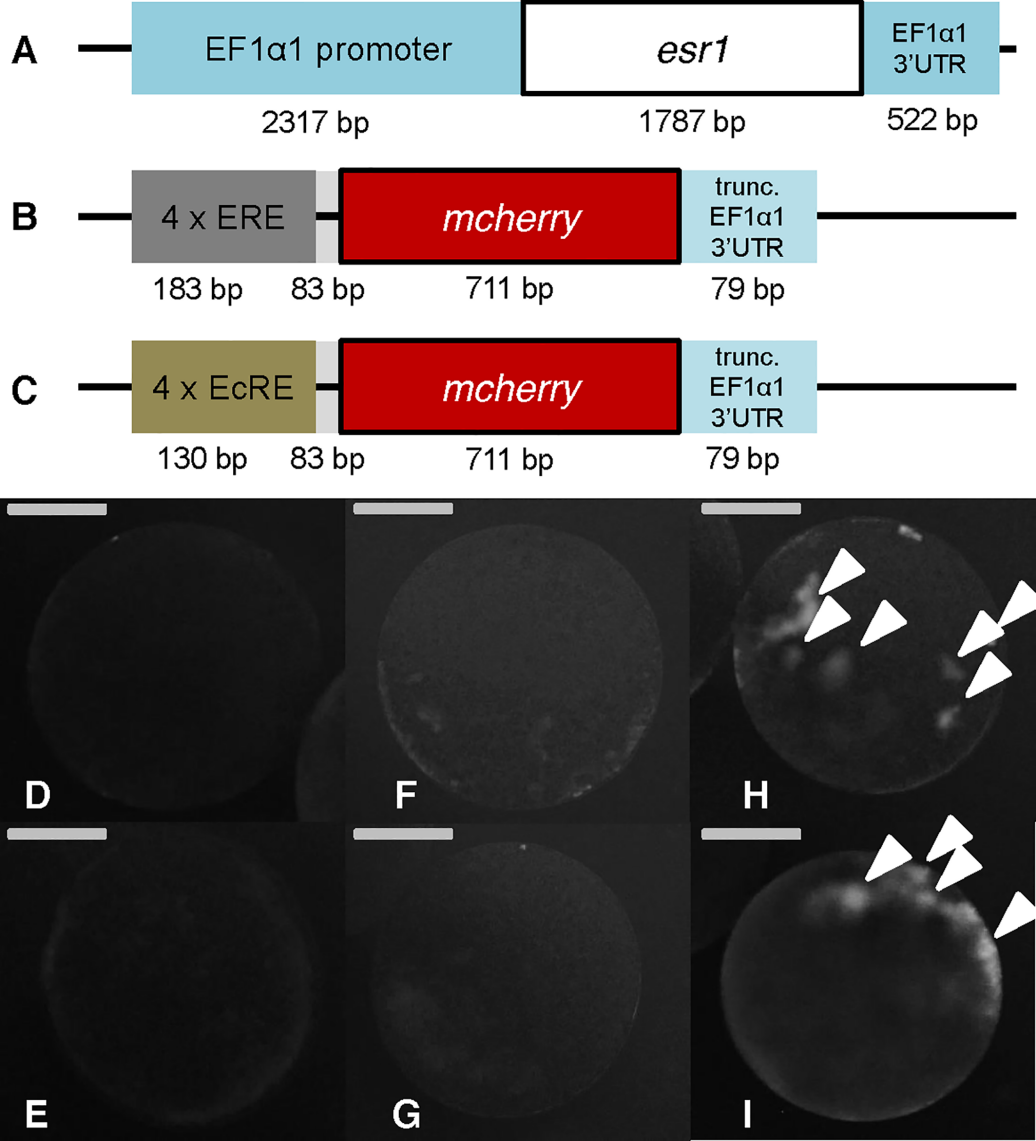
**Structure of injected plasmids (A-C) and wild type *Daphnia* embryos injected with the plasmids at 50 ng/μL each and 26.8 ng/μL (100 μM) DES, at 16 hpi (D-I).** (A) pRC21-hERa. Human estrogen receptor α (*esr1*) with EF1α1 promoter and full length EF1α1 3’UTR. (B) pRC21-ERE:mCherry. *Mcherry* with 4 x ERE repeats and a truncated version of the EF1α1 3’UTR. (C) pRC21-EcRE:mCherry. *Mcherry* with 4 x EcRE repeats and a truncated version of the EF1α1 3’UTR. Lengths of the elements (not to scale) in bp are indicated underneath. (D) negative control (uninjected), (E) injection of pRC21-hERa and pRC21-ERE:mCherry, (F) injection of pRC21-ERE:mCherry and DES, (G) injection of pRC21-hERa and DES, (H) injection of pRC21-hERa, pRC21-ERE:mCherry and DES, (I) injection of control plasmid pRC21-EcRE:mCherry. Successful activation of the reporter is marked with white arrowheads. Bar = 100 μm.

The genetic sequence for genomic integration was prepared on two separate DNA plasmids. One expresses the human estrogen receptor α (hERα) (Fig 1A) ubiquitously. The other plasmid (Fig 1B) contains 4x repeats of the Estrogen Response Element (ERE), *mCherry* as a visible reporter, and the *EF1α1* 3’UTR truncated to 60 bp plus its poly(A) signal for higher RNA stability and/or translation efficiency [8]. The sequence between the ERE repeats and the *mCherry* start codon is the same as that between the EcRE (Ecdysteroid response element) repeats and its reporter start codon in a previously established ecdysteroid reporter in this species [9]. The 4xEcRE reporter was used as a positive control in this experiment (Fig 1C).

To test the functionality of these plasmids, microinjection was conducted with wild-type (NIES) *Daphnia* eggs. Fifty ng/μL pRC21-hERa plasmid, 50 ng/μL pRC21-ERE:mCherry plasmid and 26.8 ng/μL (100 μM) DES were injected as single solution, in combinations of two or all three together. When only 1/3rd (data not shown) or 2/3rd of the components were injected (Fig 1E–1G), no red fluorescence could be detected after 18 h, similar to uninjected control eggs (Fig 1D). When injecting the two plasmids together with an estrogenic compound, DES, on the other hand, fluorescence could be detected (Fig 1H), as well as after injection of the pRC21-EcRE:mCherry control plasmid that responds to endogenous ecdysteroids (Fig 1I). Thus hERα is functional and active in an estrogen-dependent manner in *D*. *magna* embryos. We also concluded that neither hERα nor ERE are activated by endogenous compounds in *D*. *magna* eggs of this stage.

For genomic integration, we targeted the *dsred2* locus of a previously generated transgenic animal [6]. To make the donor plasmid DNA, the hERα expression cassette and ERE reporter gene were cloned into a single plasmid with the target site of TALENs that cleave the *dsRed2* gene [6]. Integration of the donor DNA into the *DsRed2* locus causes the loss of red fluorescence in absence of estrogenic compounds. We injected this donor plasmid (25 ng/μL) together with *in vitro* synthesized mRNAs that code for DsRed2-targeting TALENs (250 ng/μL each). Of the 106 embryos surviving at 1 hpi, one (0.94%) that showed a loss of red fluorescence survived into adulthood. We named this transgenic animal “ES line”.

**Fig 2:**
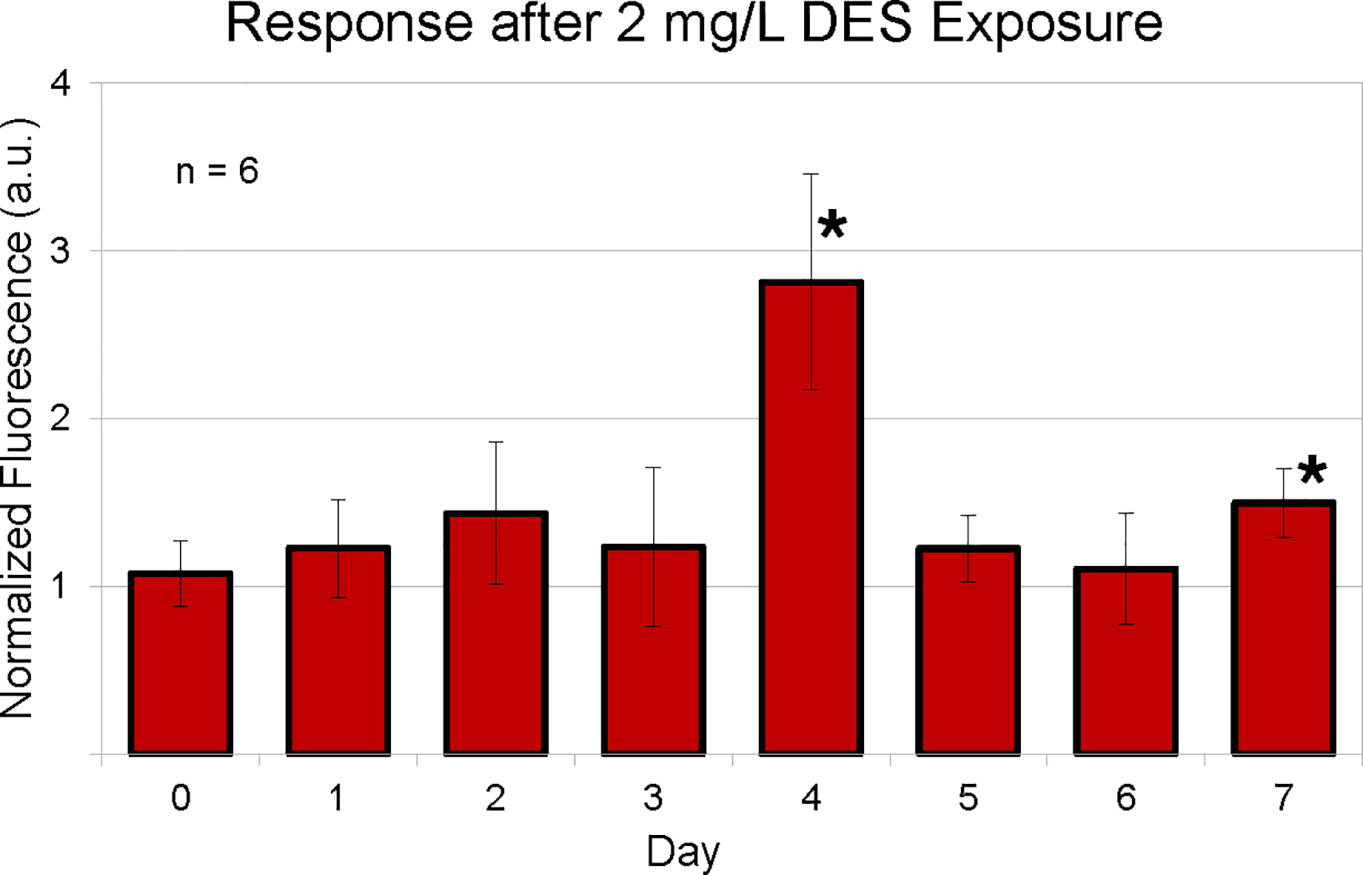
Fluorescence of thoracic appendages of ES *Daphnia* exposed to 2 mg/L DES over seven days, pictures taken every 24h. Normalized to fluorescence of control *Daphnia* of the same age, units are arbitrary (a.u.). Significant differences (*) compared to control (p<0.002) at day 4 and from day 7 onward.

**Fig 3:**
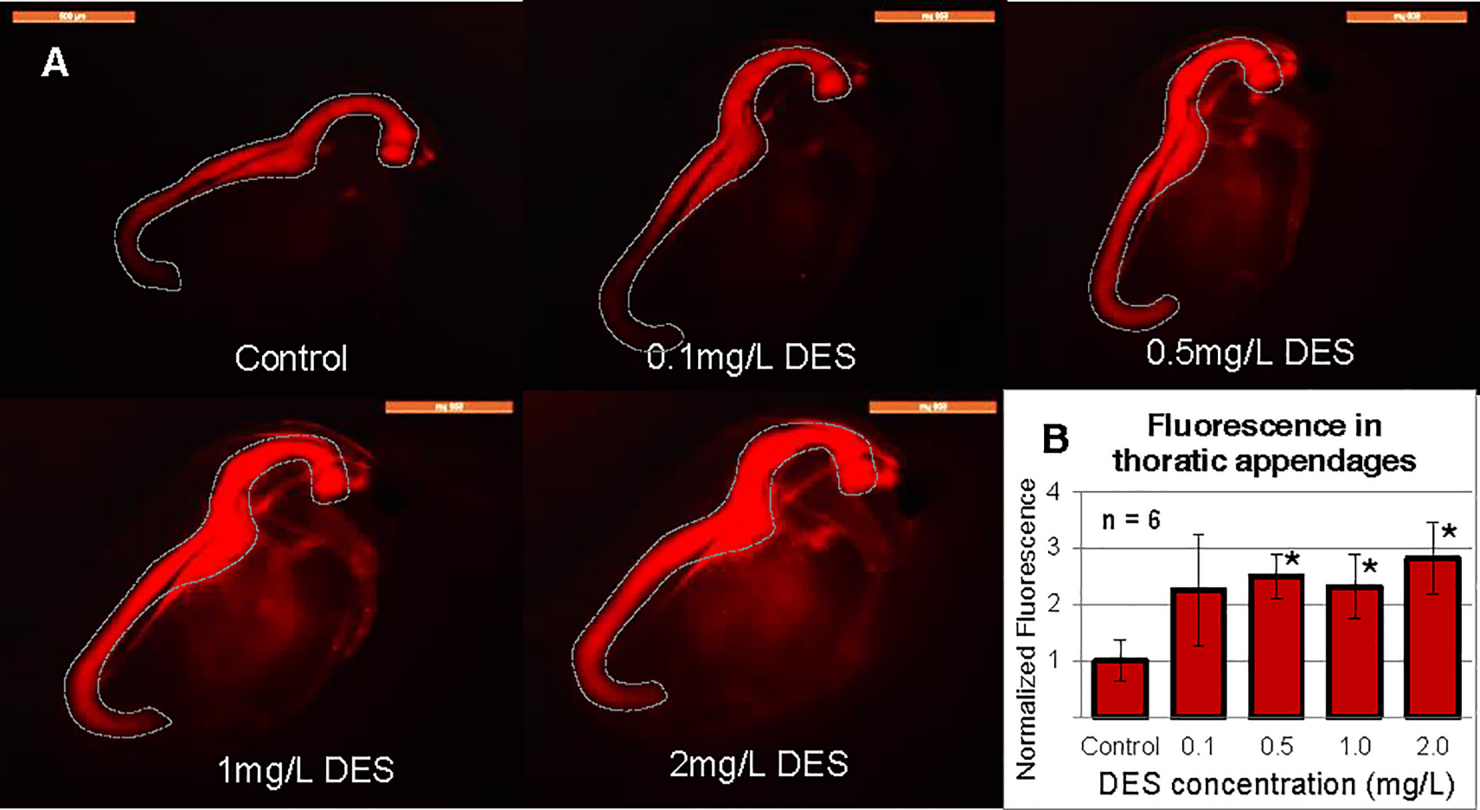
Concentration dependent response to DES. (A) ES *Daphnia* exposed to different concentrations of DES, pictures taken at day 4, bar = 100 μm. (B) Fluorescence calculated from thoracic appendages, normalized to control *Daphnia* of the same age. Asterisks (*) indicating p<0.002 compared to control. Dotted lines marking the gut (autofluorescence from fed algae).

**Fig 4:**
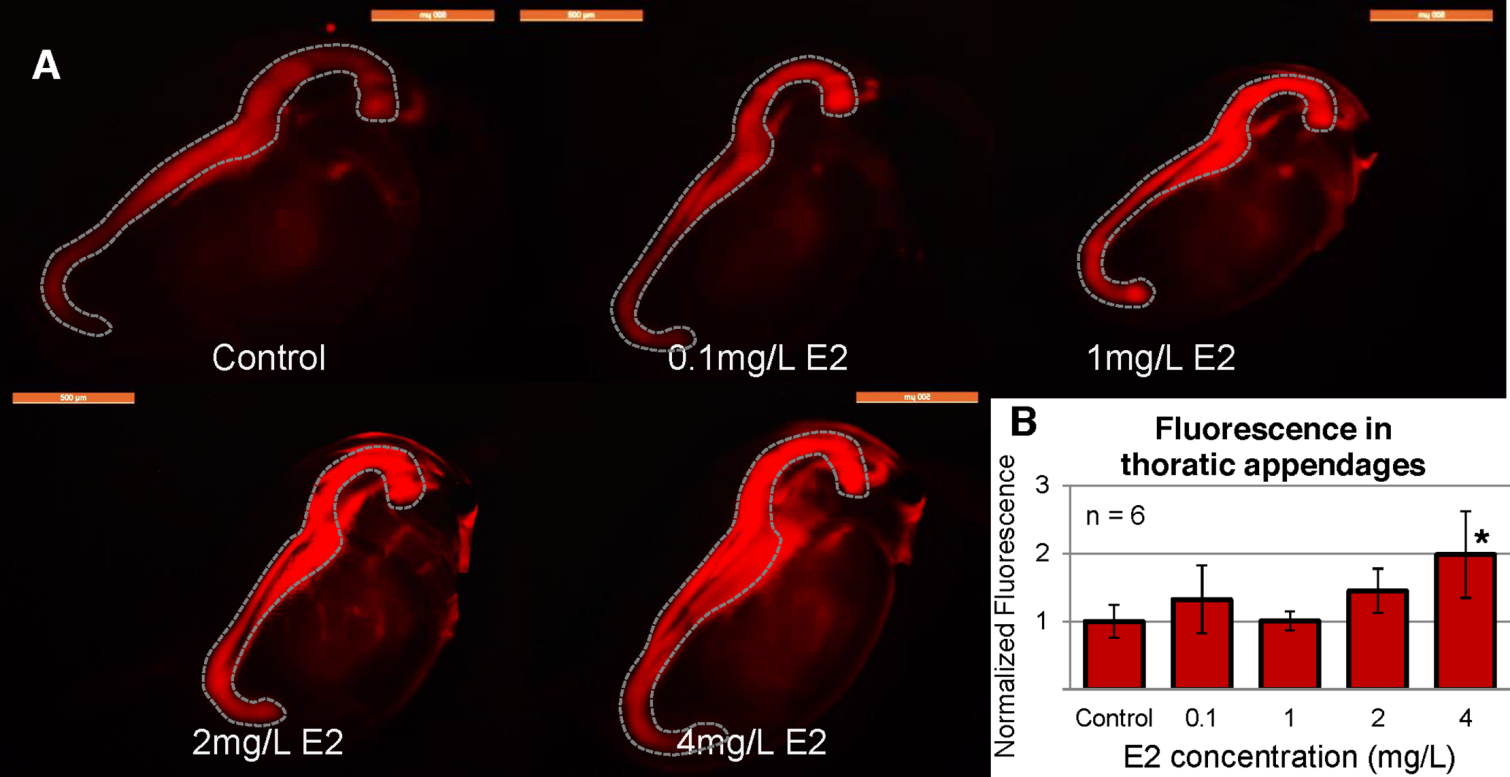
Concentration dependent response to E2. (A) ES *Daphnia* exposed to different concentrations of E2, pictures taken at day 4, bar = 100 μm. (B) Fluorescence calculated from thoracic appendages, normalized to control *Daphnia* of the same age. Asterisks (*) indicating p<0.002 compared to control. Dotted lines marking the gut (autofluorescence from fed algae).

We then exposed neonate *Daphnia* of this ES line (under 24 h old) to 2 mg/L DES for seven days to find the optimal time-point for exposure evaluation. According to the values of total fluorescence of the thoracic appendages, day four showed the highest difference between control and exposed individuals (Fig 2) so all later exposures were conducted for four days. When testing this line for sensitivity regarding DES and E2, we exposed them to 0.1 mg/L– 2.0 mg/L DES and 0.1 mg/L– 4.0 mg/L E2. In these experiments, the detection threshold for DES was 0.5 mg/L and 4 mg/L for E2 (see Fig 3, Fig 4).

## Discussion

The new estrogen responsive line of transgenic ES *D*. *magna* could successfully detect both DES and E2 at different concentrations and with different sensitivity, in accordance with their different binding affinities to hERα [16]. There was no fluorescence response without any exposure of estrogenic compounds even though the control EcRE-reporter gene [9] could detect endogenous ecdysteroids (Fig 1). The general background fluorescence in the transgenic *Daphnia* was relatively low so it is sensitive enough to detect estrogenic chemicals four days after exposure (Fig 2). This result is consistent with previous finding that *Daphnia* do not have any endogenous estrogens nor any ERs [17]. This study is the pioneering demonstration that hERα can function in *Daphnia in vivo*.

However it was notable that sensitivity of the transgenic *Daphnia* to estrogen was ranging in the low mg/L. Compared to existing biosensors for estrogen like zebrafish and medaka [18,19] or yeast, bacteria, and mammalian cell cultures [20–22], the sensitivity of this line was quite low. One of the future perspective of the use of the estrogen sensing *Daphna* may be an application to environment assessment or monitoring. In order to detect low concentration of estrogens in the ng/L ranges as a biosensor, which is where serious environmental effects can already occur [23–25], further improvement is necessary. This sensitivity could be improved by improving several points. One of the major problem may be a cofactor which bridges estrogen receptor and transcriptional machinery. In order to monitor Daphnia nuclear receptor (ecdysone receptor) function in mammalian cells, we have introduced *Taiman* as a cofactor, which suggested that direct interaction between daphnia nuclear receptor and mammalian transcriptional machinery is weak. Similarly interaction between human nuclear receptor and daphnia transcriptional machinery may be weak and introduction of a cofactor may improve its sensitivity. It may be also useful for testing further iterations of the sequence between ERE and the mCherry start codon [26], by optimizing codon usage for *Daphnia* [27–29], or by integration into a different genetic location.

Red fluorescence was expected and found all over the body of the exposed waterfleas, with stronger fluorescence in digestive tissues from feeding and the hepatopancreas which is involved in sequestering hormones taken up by feeding [30]. Stronger fluorescence was also detected in the “joints” of secondary antennae which are responsible for locomotion.

To express hERα we used the promoter of *D*. *magna EF1a1* which produces highly abundant mRNA in this species [31], which could easily lead to over-expression of hERα. And indeed reproduction was slowed down in the ES line compared to both a wild-type strain of *D*. *magna* (NIES) and the *dsred2* line. Therefore particularly the EF1α1:*esr1* sequence might need to be adjusted to lower expression levels by truncating the promoter sequence or by using promoters of the other ubiquitously expressed genes such as ribosomal protein L32 and β-actin, which show moderate expression compare to EF1.

Nevertheless, both hERα and ERE could be shown to be functional in *Daphnia* for the first time, suggesting a huge potential for use of *Daphnia* to study the interaction of human genes with environmental factors like the effect of EDCs in this study. This could be applied to improved biomonitoring of water quality, or even to screen potentially useful chemicals.

*Daphnia* cells can conduct the post-translational modifications necessary for formation of active hERα [32], as shown by the estrogen-dependent fluorescence response of ES line *Daphnia* from both active hERα and its successful activation of the ERE. These results open up the possibilities to test not only single receptors or other genes in *Daphnia*, but partial or even whole pathways for environmental effects on them, especially with still improving methods for genome editing in this species that allow for larger and larger sizes of inserted DNA [10,33].

It would be interesting to cross this line with other transgenic lines of *Daphnia* and visualize more than one stressor at a time. And to increase ease of use, it would be beneficial to implement a reporter that is not based in fluorescence but that can be detected by the naked eye or a simple light microscope. Over-expression of hemoglobin and the subsequent redder color of *D*. *magna* [34,35] is a promising approach, as is black color from expressing melanin or other darker pigments which are usually only found in *Daphnia* species exposed to higher UV radiation like *Daphnia melanica* [36,37].

In conclusion, for the first time, a human gene (*esr1*) was successfully and stably expressed in the crustacean *D*. *magna*. The resulting new transgenic line of *D*. *magna* could indicate presence of both DES and E2 after exposure in a dose-dependent manner.

## Materials and methods

### *Daphnia* strain

A transgenic line of *Daphnia magna* containing a hemizygous *DsRed2* gene under the control of the *D*. *magna EF1α-1* promoter was generated previously [6] from a *D*. *magna* NIES clone (obtained from the National Institute for Environmental Studies, NIES; Tsukuba, Japan). This *dsred2* line has been maintained for more than 50 generations. It exhibits ubiquitous *DsRed2* expression.

### *Daphnia* culture conditions

Eighty neonates of *dsred2 Daphnia* (under 24 h old) were cultured in 5 L of the Aachener Daphnien Medium (AdaM) [38] at 22–24°C under a light/dark cycle of 16/8 h. The culture medium was renewed once a week. *Daphniids* were fed every day with 5 mg of *Chlorella vulgaris* (Nikkai Center, Tokyo, Japan) during the first week. After maturation, offspring was removed daily and adults were fed with 10 mg *Chlorella* per day.

After establishment of the estrogen responsive line (ES line), ES *daphnia* were cultured under the same conditions with the exception of feeding and juvenile removal. ES *daphniids* were fed every other day with 6 mg of *Chlorella vulgaris* (Nikkai Center, Tokyo, Japan) until maturation. Then, offspring was removed twice a week and adults were fed with 10 mg *Chlorella* every other day.

All methods regarding animal use were carried out in accordance with the relevant guidelines and regulations.

### Microinjection

Microinjection was conducted according to established procedures [39]. In short, adult *D*. *magna* (*dsred2* line) with empty brood chambers were selected and observed until ovulation started. Then *D*. *magna* were transferred to ice-chilled M4 medium [40] containing 80 mM sucrose (M4-suc) and dissected to collect the eggs within the brood chamber. The eggs were stored in ice-chilled M4-suc medium until injection to slow the hardening of the egg membrane. Microinjection was performed within 1 hour post ovulation (hpi) for the same reason. Successfully manipulated eggs were transferred to fresh medium and were cultured in a 96-well plate at 23 ± 1°C. From each clutch of eggs, 2–3 eggs that were not injected served as control for development.

### Plasmid construction

To generate the hERα expression plasmid, full length *D*. *magna EF1α-1* promoter [29] and full length human *esr1* [41] were joined into a pRC21 backbone with full length *EF1α-1* 3’UTR via InFusion (TAKARA, Kusatsu, Shiga, Japan); the resulting construct was termed pRC21-hERa.

To generate the EcRE reporter plasmid, full length *mCherry* and the first 60 bp of *EF1α-1* 3’UTR [8] were joined into a pRC21 backbone containing the last 13bp of *EF1α-1* 3’UTR and therefore providing a poly(A) signal via InFusion (TAKARA). A 4xEcRE promoter [9] was amplified via polymerase chain reaction (PCR) with primers introducing a restriction site for MscI. Both the PCR fragment and the *mCherry* plasmid were digested with MscI and EcoO109I (NewEngland BioLabs, Ipswitch, MA, USA) and joined via MightyMix ligation (TAKARA); the resulting construct was termed pRC21-EcRE:mCherry.

To generate the ERE reporter plasmid, the EcRE repeats were excised out of pRC21-EcRE:mCherry with EcoO109I and XmaI (NewEngland BioLabs). A 4xERE sequence [42] was amplified with primers introducing a restriction site for XmaI, it contains an EcoO109I site. This PCR fragment was also digested with EcoO109I and XmaI (NewEngland BioLabs) and joined into the backbone plasmid via MightyMix ligation (TAKARA); the resulting construct was termed pRC21-ERE:mCherry.

For genomic integration, pRC21-hERa as the backbone was digested with SalI and NdeI (NewEngland BioLabs). pRC21-ERE:mCherry was digested with BssHII and NdeI (NewEngland BioLabs). The *dsred2* TALE site was amplified from genomic DNA with PCR primers introducing digestion sites for SalI and BssHII. After digestion, all three fragments were joined via MightyMix ligation (TAKARA); in the resulting construct the ERE reporter and the ER halves face opposite directions, it was termed pRC21-estrogensensor.

All plasmids were transformed into XL10-GOLD *E*. *coli* after ligation, harvested with a PureYield Miniprep kit (PROMEGA, Fitchburg, WI, USA), purified by phenol/chloroform extraction followed by ethanol precipitation and their sequence was confirmed by sequence analysis.

### *In vitro* RNA synthesis

For TALEN mRNA synthesis, left and right TALEN expression plasmids [6] were linearized with Acc65I (NewEngland BioLabs), and purified using the QIAquick PCR purification kit (QIAGEN GmbH, Hilden, Germany). Linearized DNA fragments were used for *in vitro* transcription with the mMessage mMachine kit (Life Technologies, CA, USA). Poly(A) tails were attached to TALEN RNAs using a Poly(A) Tailing Kit (Life Technologies), following the manufacturer’s instructions. The synthesized RNAs were column purified using the Rneasy Mini Kit (QIAGEN GmbH, Hilden, Germany), followed by phenol/chloroform extraction, ethanol precipitation, and resuspension in DNase/RNase-free ultra pure water (Life Technologies).

### Exposure

Diethylstilbestrol (DES) (Sigma Aldrich, St. Louis, MO USA) and 17β-Estradiol (E2) (Sigma Aldrich) were dissolved in 100% *N*,*N*-Dimethylformamide (DMF) (Nacalai tesque, Kyoto, Japan) to final concentrations of 10 mg/mL as stock solutions. For exposure, the stock was diluted with AdaM [36] to final solvent concentrations of under 0.2%. For exposure, neonate *daphniids* (under 24 h old) were kept in 24 well plates (Thermo Fisher Scientific, Waltham, MA USA), one individual in 2 mL medium per well, at 23 ± 1°C under a light/dark cycle of 16/8 h, with medium renewal every day.

### Fluorescence microscopy

*Daphnia* were partially immobilized in minimal amounts of medium on micro slide glasses (Matsunami, Osaka, Japan). Red fluorescence intensity was recorded with a color digital camera (Leica DC500) mounted on a Leica M165C fluorescence microscope (Leica Microsystems Heidelberg GmbH, Mannheim, Germany) equipped with a 545 nm excitation and a 620 nm barrier filter. The pictures were taken under 63 × magnification with 100% aperture, 1 s exposure, 3.0 gain, 1.5 saturation, and 1.0 gamma. The red fluorescence intensity of neonates was recorded every 24 h in the first exposure experiment, at day 4 of exposure henceforward.

### Fluorescence quantification

Fluorescence intensity (fluo) was quantified using ImageJ software. Previously reported methods [8,43,44] were adapted to reduce background interference. In this study, only the area of thoracic appendages (thorap) was used for calculations with the following formula.

Fluo(thorap)=totalfluorescenceofthoracicappendages−(numberofpixelsoftheselectedarea×meanofthreebackgroundfluorescencemeasurements)

This value was then normalized for exposed *Daphnia* by defining the value of unexposed *Daphnia* of the same age as 1.
